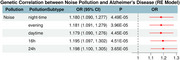# Impact of Noise Pollution on Alzheimer's Disease: Insights from Polygenic Scores and Mendelian Randomization Analysis

**DOI:** 10.1002/alz70855_107145

**Published:** 2025-12-25

**Authors:** Jingchun Chen, Faria Tavacoli, Hayley Ho, Alice Lee

**Affiliations:** ^1^ University of Nevada Las Vegas, Las Vegas, NV, USA

## Abstract

**Background:**

Epidemiological evidence suggests a link between noise pollution exposure and Alzheimer's disease (AD); however, its causal relationship remains unclear. This study aimed to evaluate the potential genetic correlation and causal association between noise exposure and AD risk using comprehensive genetic approaches.

**Method:**

Genome‐wide association study (GWAS) data for noise pollution from the UK Biobank were used to calculate polygenic scores (PGSs) in AD cases and controls. Logistic regression analyses were conducted in the discovery cohort (ADc1234ADA: 2,651 cases, 2,768 controls) and the replication cohort (ADNI: 679 cases, 645 controls) using PRSice‐2 software. The PGS associations with AD were adjusted for covariates including sex, age, and APOE ε4 allele count. Meta‐analyses were performed across datasets, with Bonferroni‐corrected *p*‐values < 0.05 considered significant. Mendelian Randomization (MR) analyses using the inverse‐variance weighted (IVW) method further evaluated the causal relationship.

**Result:**

PGSs for noise pollution at average 24‐hour sound levels were significantly associated with AD risk (OR = 1.198; 95% CI: 1.100–1.235; *p* = 3.65 × 10⁻⁵) from the meta‐analysis. MR analyses indicated that genetically predicted frequent exposure to very noisy workplaces was associated with a higher risk of AD (OR = 3.251; 95% CI: 1.645–7.214; *p* < 0.004) and a lower likelihood of longevity (OR = 0.635; 95% CI: 0.500–0.806; *p* < 1.92 × 10⁻⁴). Sensitivity tests supported these causal effects.

**Conclusion:**

This study provides robust evidence supporting a causal link between noise pollution and AD risk. The findings highlight the importance of reducing noise exposure, particularly in noisy workplaces, as a public health strategy to lower AD risk and enhance longevity.